# Innexin Expression in Electrically Coupled Motor Circuits✩

**DOI:** 10.1016/j.neulet.2017.07.016

**Published:** 2017-07-13

**Authors:** Adriane G. Otopalik, Brian Lane, David J. Schulz, Eve Marder

**Affiliations:** a Volen Center and Biology Department, Brandeis University, Waltham, MA 02454, USA; b Division of Biological Sciences, University of Missouri-Columbia, Columbia, MO, 65211, USA

**Keywords:** Stomatogastric ganglion, Cardiac ganglion, Crustacean, Central pattern generators, Neuronal homeostasis

## Abstract

The many roles of innexins, the molecules that form gap junctions in invertebrates, have been explored in numerous species. Here, we present a summary of innexin expression and function in two small, central pattern generating circuits found in crustaceans: the stomatogastric ganglion and the cardiac ganglion. The two ganglia express multiple innexin genes, exhibit varying combinations of symmetrical and rectifying gap junctions, as well as gap junctions within and across different cell types. Past studies have revealed correlations in ion channel and innexin expression in coupled neurons, as well as intriguing functional relationships between ion channel conductances and electrical coupling. Together, these studies suggest a putative role for innexins in correlating activity between coupled neurons at the levels of gene expression and physiological activity during development and in the adult animal.

## 1. Introduction

Innexins are the molecules that form gap junctions in invertebrates [1,2]. Over the past 20 years, innexin genes have been identified in many invertebrate species and it is now clear that, like the vertebrate connexins, there are many different innexin genes in the numerous species in which they have been characterized [2–4]. Much of the work on innexins has focused on their roles in development [5–8] and on their possible membrane signaling roles unrelated to gap junction formation [9,10].

Furshpan and Potter's seminal demonstration of electrical coupling in the giant fiber system of crayfish [11] was notable because this first study of electrical coupling was of a rectifying junction, one that passes current preferentially in one direction [12–14]. Molecular work on the giant fiber system in *Drosophila* has suggested that rectification is a consequence of differential expression of innexin isoforms on the two sides of the junction [15], and expression studies of *Drosophila* innexin genes in oocytes showed that the electrophysiological properties of the junctions depend on the particular subset of innexins expressed [16].

These data pose a specific set of fundamental questions in understanding how electrical coupling strength and rectification are determined in functional neuronal circuits. Here, we review what is known about electrical coupling and innexin expression in two small crustacean rhythmic central pattern generators, the stomatogastric ganglion and the cardiac ganglion. In both of these small ganglia, as is typical for many circuits, electrical coupling is both between neurons of the same cell type and between different cell types [17–19].

## 2. Overview of the connectivity of the stomatogastric ganglion

**Fig. 1:**
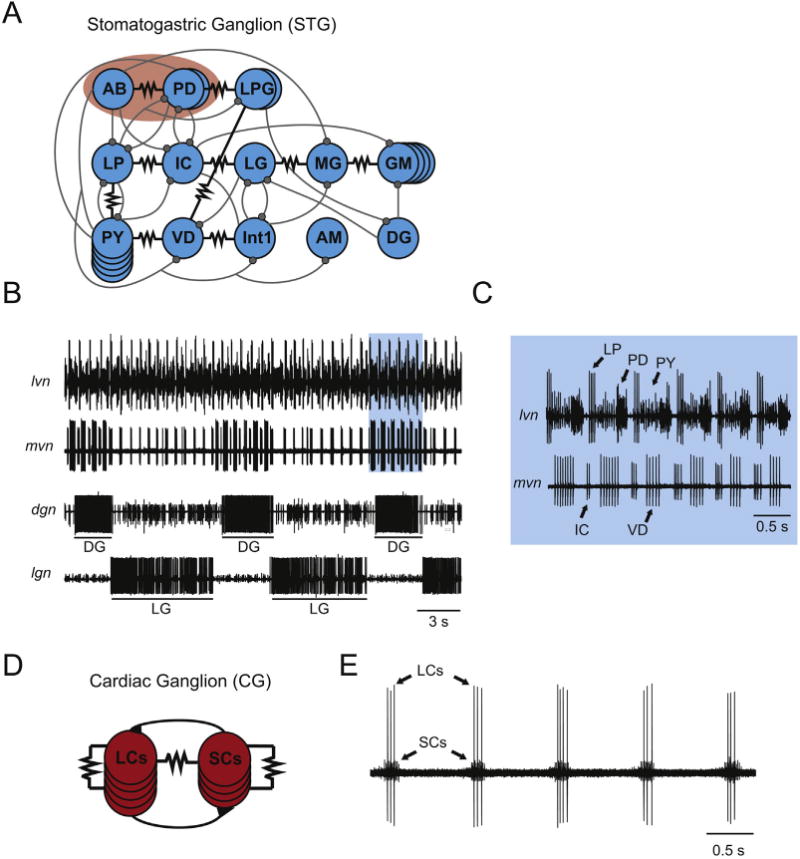
Connectivity and Motor Output of Two Crustacean Central Pattern Generators. A. Connectivity diagram of the crustacean stomatogastric ganglion (STG) with inhibitory chemical synapses indicated in gray circles and electrical synapses indicated with black resistor symbols. The pacemaker kernel, consisting of 1 Anterior Burster (AB) interneuron and 2 Pyloric Dilator (PD) motor neurons is indicated with red shading. B and C. Extracellular nerve recordings of the lateral ventricular (lvn), medial ventricular (mvn), dorsal gastric (dgn), and lateral gastric (lgn) nerves show the relative firing and phase relationships between various stomatogastric ganglion neurons. Recordings show simultaneous activation of the fast pyloric rhythm (indicated in blue shading in (B) and expanded in C): involving the Lateral Pyloric (LP), Pyloric (PY), Inferior Cardiac (IC), Ventricular Dilator (VD), and PD neurons, as well as the slow gastric mill rhythm: involving the Dorsal Gastric (DG) and Lateral Gastric (LG) neurons. D. Connectivity diagram of crustacean cardiac ganglion (CG) with excitatory chemical synapses indicated with triangles and electrical synapses indicated with black resistor symbols. Small cell pacemakers (SCs) drive burst rate and duration via excitatory inputs to Large Cells (LCs), while LCs provide feedback to SCs via excitatory synaptic and/or electrotonic connections to prolong burst duration. E. Extracellular nerve recording from the cardiac ganglion showing the rhythmic output of SCs and LCs, which are easily distinguished by spike amplitude.

The stomatogastric ganglion contains 26–30 neurons (depending on species) and produces two rhythmic motor patterns, the fast pyloric rhythm (period ∼1 s) and the slower gastric mill rhythm (period ∼5–15 s) [20]. These rhythms are responsible for moving two regions of the crustacean stomach [21]. Fig. 1A shows the connectivity diagram of the stomatogastric ganglion of the crab, *Cancer borealis*, with the electrical synapses (black resistor symbols) shown more prominently than the chemical inhibitory synapses (shown as gray circles). Several cell types are found in multiple copies: in *C. borealis* there are two PD (Pyloric Dilator) neurons, two LPG (Lateral Posterior Gastric) neurons, five PY (Pyloric) neurons, and four GM (Gastric Mill) neurons [22]. While the pairs of PD and LPG neurons are strongly electrically coupled [4], the PY and GM neurons are less strongly electrically coupled. There are numerous instances in which neurons of different cell types are electrically coupled. Most notably, the single AB (Anterior Burster) neuron is strongly electrically coupled to the PD neurons, and the strong coupling between the AB and two PD neurons creates a synchronous pattern of discharge that serves as the pacemaker kernel for the pyloric rhythm [23,24].

Most of the electrical synapses shown in Fig. 1A are between neurons of different cell types, considerably weaker than those coupling the PD and AB neurons, and rectifying [4]. Also, there are numerous examples of neurons that are electrically coupled and fire out of phase with each other, or even in different rhythms [17]. The extracellular recordings in Fig. 1B show the pyloric and gastric rhythms produced by the stomatogastric ganglion. Fig. 1C is an expanded view of the blue box highlighted in Fig. 1B and shows the relative timing of the pyloric network neurons. Fig. 1A shows electrical synapses between VD (Ventricular Dilator), Int 1 (Interneuron 1), PY and LPG. The recordings in Fig. 1C show that VD is firing in phase with PY, but the LPG neurons often fire with the PD neurons, and Int 1 and LG (Lateral Gastric) are active in the gastric mill rhythm.

The connectivity diagram in the stomatogastric ganglion highlights an important conceptual and technical difficulty: most stomatogastric ganglion neurons are electrically coupled to more than one other type of neuron. This means that it is difficult to know how the expression of specific innexin genes maps onto the physiological properties of specific electrical synapses between any two neurons [4]. We expect that whenever neurons are electrically coupled to more than one other type of neuron, this may confound the analysis of the roles of different innexins in these junctions.

The widespread electrical coupling of the stomatogastric ganglion (Fig. 1) has another consequence: it creates many “parallel pathways” in which a presynaptic neuron can influence a given postsynaptic neuron by more than one route [17,14,25,26,22]. Such parallel pathways create the opportunity to produce similar changes in circuit output via different mechanisms [25].

## 3. Overview of the connectivity of the cardiac ganglion

The crustacean heart is single-chambered and each heartbeat is triggered by bursts of activity in five electrically coupled large motor neurons in the cardiac ganglion. In turn, the large cell (LC) bursts are triggered by activity in four bursting small cell (SC) interneurons that are thought to be bursting pacemakers and to drive the LC bursts [27–29]. Fig. 1D shows the putative connectivity of the CG, with electrical coupling between the LCs, the SCs, and within the two classes of neurons. Fig. 1E shows patterns of discharge from the cardiac ganglion of *C. borealis*. The SC spikes usually start before and end after the LC spikes.

## 4. Correlations in ion channel expression in electrically coupled neurons

Because there are two, tightly electrically coupled, PD neurons in each STG, it is possible to compare the animal-to-animal variability in ion channel expression across animals with the variability across the two PD neurons within individual animals [30]. The expression of ion channel genes shows a 2–6-fold range of ion channel expression and conductance densities for many cell types and genes in both STG and CG neurons [4,30–35].

**Fig. 2:**
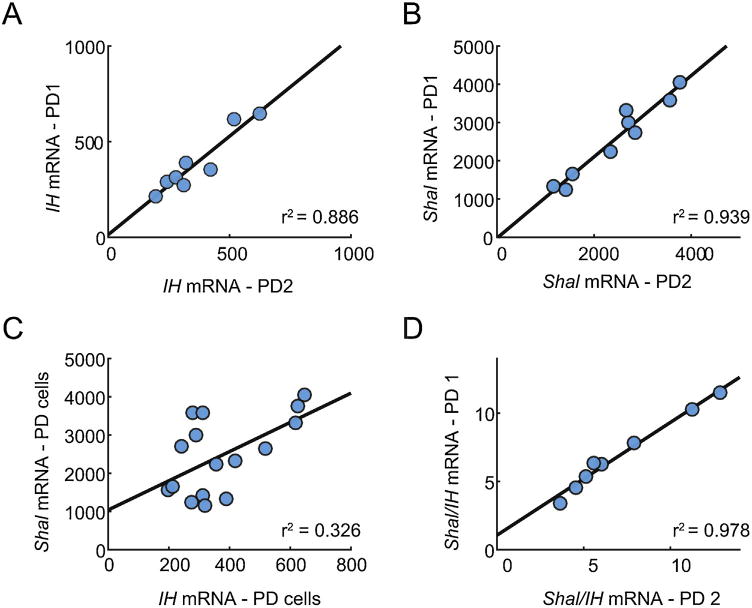
Ion channel correlations in electrically coupled PD neurons. Paired PD neurons have correlated abundances of *Shal* and *IH*. A and B. Abundances of either *IH* or *Shal* mRNA, respectively, were plotted for electrically coupled pairs of PD neurons from individual crabs (n = 8 pairs of PD neurons), with one PD neuron on the x-axis (PD1) and the other on the y-axis (PD2). C. Abundances of *Shal* versus *IH* mRNA in individual PD neurons (n = 16 from 8 animals). D. Ratios of *Shal* to *IH* mRNA for pairs of PD neurons. Lines plotted in A-D show the linear fits. The linear regression analyses yielded statistically significant positive linear correlations p values < 0.01 and good fits with relatively high r^2^ values (indicated in lower left of each plot) for A, B and D. Replotted from Schulz et al. [30].

Fig. 2A shows a plot of the mRNA copy number for the I_H_ (*HCN*) gene in the PD neurons of the crab. This plot shows a tight correlation between the I_H_ expression in the two PD neurons from the same animal. Although there was a 3–4-fold range of values across animals, the tight correlation shows that the two, tightly coupled PD neurons in the same animal showed very similar mRNA expression levels [30]. Fig. 2B shows similar data for the *shal* K^+^ channel gene that encodes for A-type currents in STG cells [36]. Interestingly, when the *I_H_* and *shal* levels were plotted against each other in the entire data set (Fig. 2C), there was only a weak correlation between their levels. However, when we plot the *shal*/*I_H_* ratio in the two PD neurons from each ganglion, we obtain a very strong correlation (Fig. 2D). Together, these data show that these two conductances show highly correlated expression in PD and other neurons [30,31], and the expression of these genes in the tightly electrically coupled neurons is extremely closely matched.

Correlations in the expression of ion channel conductances arise automatically in homeostatic models of intrinsic excitability in single neurons [37–39], where specific sets of correlations are associated with characteristic patterns of intrinsic excitability. It is interesting to speculate that any substances that cross the electrical junctions and influence channel expression might also tend to keep the electrically coupled neurons more similar in conductance densities than might otherwise occur.

## 5. Electrical coupling in network stability and homeostasis

**Fig. 3:**
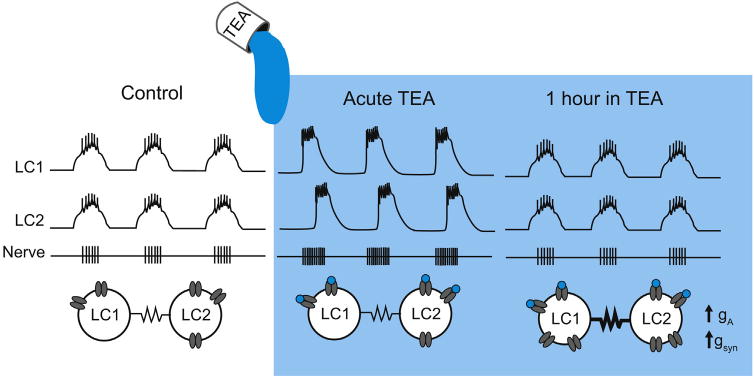
Homeostatic increase in electrical synaptic strength restores synchrony in the cardiac ganglion following administration of TEA. Cartoon representation of the results in Lane et al. [42]. Schematic of intracellular voltage waveforms and extracellular nerve recordings demonstrating the activities of two representative electrically-coupled large cells (LCs) in control (left), acute, and long-term tetraethylammonium (TEA) application. Below: simplified circuit diagrams demonstrating changes in intrinsic and synaptic properties with TEA application. LC1 and LC2 burst synchronously under control conditions. Acute administration of 25 mM TEA (blue) partially blocks high-threshold voltage-gated potassium channels (in gray) and leads to hyperexcitability in the cardiac ganglion and partial desynchronization of LC slow-waves. After 1 h of TEA application the LC slow-wave synchrony is restored and can be attributed to complementary compensatory increases in the A-type potassium conductance (g_A_) and electrical synapse strength (g_syn_).

Electrical coupling can vary in response to neuromodulation or network activity [40,41]. The plasticity of electrical synapses provides a robust mechanism for network stability or synchronization in the face of differing synaptic inputs, heterogeneous modulatory projections, or perturbations that may otherwise desynchronize pairs of neurons. Recent work in the cardiac ganglion demonstrated a role for electrical synapses in homeostatic recovery of network synchrony in response to application of the high-threshold potassium channel blocker tetraethylammonium (TEA) [42]. Application of TEA results in the temporary loss of coordinated motor output among large cells (Fig. 3). Interestingly, the network compensates for this desynchronizing perturbation within one hour. The authors demonstrate that restoration of large cell synchrony depends on at least two processes: alteration of one of the potassium currents and increases in the electrical coupling between the large cells [42]. This is an example of a homeostatic compensatory process that depends on at least two changes in circuit parameters to bring the circuit back to its control activity pattern.

Lane et al. [42] reveal a curious synergy between electrical coupling and ion channel conductances in stabilizing cardiac ganglion output. Neurons of the cardiac ganglion express correlated ion channels abundances [32] and coregulation of ion channel conductances preserves motor output [43]. This raises the question of whether electrical coupling may endow coupled neurons with the shared small molecules or physiological activity to promote such ion channel correlations in this small motor circuit.

## 6. Correlations in innexin expression in circuit neurons

**Fig. 4:**
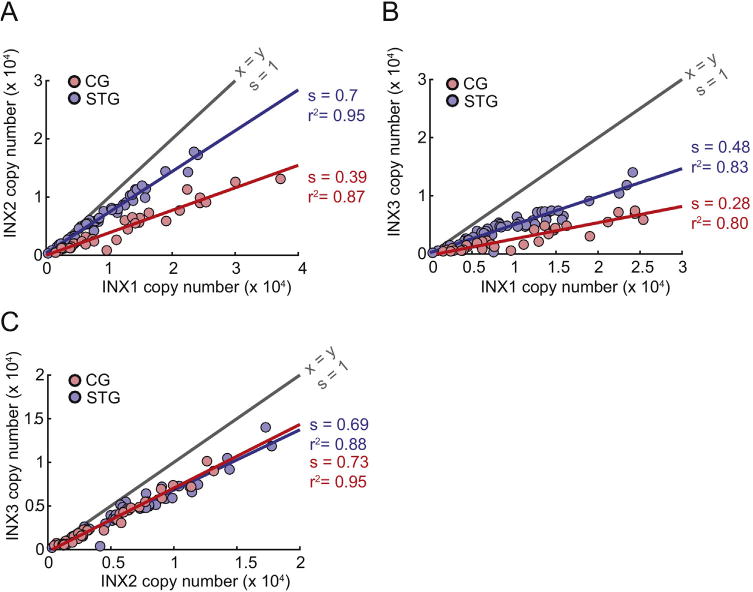
Correlated Innexin abundances in two small neuronal circuits. mRNA abundances for innexin 1 (INX1), innexin 2 (INX2), and innexin 3 (INX3) were acquired for various single identified neurons from the stomatogastric ganglion (STG; n = 34 neurons from 9 animals, including: 6 LG neurons, 11 LPG neurons, 3 Medial Gastric (MG) neurons, 4 PY neurons, 5 IC neurons, 4 DG neurons, and 1 Anterior Gastric Receptor (AGR) neuron) and 49 LCs from 10 cardiac ganglia. A. Abundances of INX2 versus INX1. B. Abundances of INX3 versus INX 1. C. Abundances of INX3 versus INX2. A-C. mRNA abundances from single STG neurons are plotted in blue, and those for CG neurons are in red. Gray line shows the identity line. Red and blue lines are the linear fits for CG and STG data, respectively. The slopes (s) and r^2^ values are indicated on the right of each curve. In all cases, linear regression analyses yielded p < 0.01. Methods as in Tobin et al. [32] and Temporal et al. [34].

The first molecular studies of the innexins in crustaceans were done in adult and developing STG of the lobster *Homarus gammarus* [6]. These authors identified two innexin genes and correlated their expression with the development of the animal. Subsequent work shows that there are at least 6 innexin genes in the nervous systems of *C. borealis* and *Homarus americanus* [4]. Many STG neurons express at least *INX1*, *INX2*, and *INX3*. Moreover, there are strong correlations between the expression of these genes in individual STG and CG neurons when these genes are assayed at the single neuron level (Fig. 4).

Similar innexin expression among coupled neurons may promote the connectivity of these neurons during development. Work in the nervous system of the medicinal leech *Hirudo verbana* demonstrates shared innexin expression in coupled neuronal networks. There are 15 innexin genes expressed in the *Hirudo* nervous system and the identifiable neuron types express different subsets of innexins [3]. Neurons participating in the same coupled networks have shared innexin expression. Interestingly, ectopic expression of a shared innexin gene in a non-coupled cell during development can result in the inclusion of that neuron in the coupled network [8]. Taken together, this work suggests that similar innexin expression plays a role in coupling the correct neuronal pairs and determining circuit connectivity during development. Consequently, future work in the stomatogastric and cardiac ganglia may seek to better understand the combinatorial nature of innexin expression across different cell types and how specific innexin genes may promote the circuit connectivity during development.

## 7. Conclusions

The crustacean stomatogastric and cardiac ganglia are two small central pattern generating circuits endowed with numerous electrical synapses. Electrophysiological, molecular, and computational studies reveal intriguing correlations in ion channel and innexin expression in coupled neurons, as well as intriguing functional relationships between ion channel conductances and electrical coupling. These studies lay the groundwork for better understanding the roles of innexins in maintaining stable network output in the adult animal and promoting circuit connectivity during development.

## Highlights

- The crustacean stomatogastric and cardiac ganglia express multiple innexins.
- Innexins play diverse roles during development and in network homeostasis.
- Electrically coupled neurons show correlated ion channel and innexin expressions.
- Innexins may correlate activity between coupled neurons via multiple mechanisms.
